# Supplementary material for: Morphology and Vessel Density of the Macula in Preterm Children Using Optical Coherence Tomography Angiography
Source: J Clin Med. 2022 Feb 28;11(5):1337. doi: 10.3390/jcm11051337 (PMC8911277; doi:10.3390/jcm11051337)
Supplement: Supplementary file 1 [file jcm-11-01337-s001.zip › jcm-1574815-supplementary.pdf]

Table S1. Clinical and ophthalmic characteristics of preterm children – comparison between analyzed eyes and eyes excluded from the analysis. Data are presented as a number (%) or median and interquartile range (IQR).

Parameter	GCC			OCTA			CCT		
	Analyzed eyes N=203	Excluded eyes N=43	P-value	Analyzed eyes N=106	Excluded eyes N=140	P-value	Analyzed eyes N=179	Excluded eyes N=43	P-value
Age, years	10.7 (7.4-13.1)	9.5 (8.8-11.4)	0.272	12 (9.1-14.4)	9.3 (6.9-11.1)	**<0.001**	10.3 (8.3-12.7)	10.8 (7.1-13.1)	0.919
Birth weight <1500 g, N (%)	83 (40.9%)	7 (16.3%)	**0.002**	45 (42.5%)	45 (32.1%)	**0.043**	67 (37.4%)	23 (53.5%)	0.679
Gestational Age <28 weeks, N (%)	47 (23.2%)	23 (43.5%)	**<0.001**	18 (16.98%)	52 (37.1%)	**0.005**	50 (27.9%)	20 (46.5%)	0.766
Apgar score <4, N (%)	32 (15.8%)	14 (32.6%)	0.227	15 (14.2%)	31 (22.1%)	0.131	33 (18.4%)	13 (30.2%)	0.739
ROP, N (%)	98 (48.3%)	36 (83.7%)	**<0.001**	50 (47.2%)	40 (28.6%)	**<0.001**	92 (51.4%)	42 (97.7%)	0.191
ROP with treatment, N (%)	40 (19.7%)	28 (65.1%)	**<0.001**	18 (17%)	50 (35.7%)	0.544	52 (29.1%)	17 (39.5%)	0.081
Visual Acuity (logMAR)	0 (0.1- 0) N=179	0.1 (0.5-0) N=28	**0.001**	0 (0.5-0) N=93	0.1 (0.22-0) N=114	**<0.001**	0 (0.1-0) N=161	0.9 (0.6-0) N=46	0.084
Refractive error (Dioptres)	1.0 (-0.38-2.27) N=172	-3.0 (-7.25-3) N=27	**0.014**	0.5 (-0.75-1.25) N=83	1.0 (-0.87-3.0) N=116	0.122	1.0 (-0.5-2.27) N=172	-3.0 (-7.25-3.0) N=27	0.670

N – number of eys, ROP – retinopathy o prematurity, GCC – ganglion cell complex, OCTA- optical coherence tomography angiography, CCT- central choroidal thickness, p-value <0.05 are considered statistically significant
